# Biologic Therapies and Quality of Life in Pediatric Patients with Asthma: A Systematic Review

**DOI:** 10.3390/healthcare13222824

**Published:** 2025-11-07

**Authors:** Beatriz Luzio Vaz, Daniel Marrinhas, Anabela Pereira

**Affiliations:** 1 Hospital Dona Estefânia, Unidade Local de Saúde de São José, 1169-045 Lisboa, Portugal; ana.vaz3@ulssjose.min-saude.pt; 2 Center for Research in Education and Psychology (CIEP), Department of Psychology, University of Évora, 7005-345 Évora, Portugal; daniel.marrinhas@uevora.pt

**Keywords:** biologic therapies, Quality of Life (QoL), asthma, pediatric, children, adolescents

## Abstract

**Background/Objectives**: Pediatric asthma is the most prevalent chronic respiratory condition in children and adolescents worldwide and remains a major contributor to morbidity, school absenteeism, and the use of integrated healthcare services. The main goal of this systematic review is to synthesize the available evidence about the impact of biologic therapies on the quality of life in the pediatric population (children and adolescents) with asthma. **Methods**: This systematic review followed the PRISMA guidelines. A comprehensive search was performed across PubMed, Scopus, and Web of Science for articles published between 2015 and 2025 in English, Portuguese, or Spanish. Studies were eligible if they included pediatric patients (<18 years) with asthma receiving biologic therapies (e.g., omalizumab, mepolizumab, dupilumab) and reported health-related quality of life (HRQoL) outcomes using validated instruments. Article selection followed PICOS criteria and excluded reviews, case reports, and editorials. Risk of bias was assessed using the Mixed Methods Appraisal Tool. **Results**: A total of 576 articles were found and screened, and 8 studies were selected. The characteristics of the studies highlighted the involved countries, study design, number of participants, conditions, type of biologic intervention, duration, and follow-up. In addition, the importance of biologic therapies in patients’ HRQoL was presented. All eight included studies reported statistically significant improvements in HRQoL using appropriate scales. **Conclusions**: The studies reinforced the importance of biologic therapies to improve HRQoL in both patients and families/caregivers. Implications for health promotion, in particular, greater involvement of integrated healthcare comprising health professionals, family, school contexts, and the community, are discussed.

## 1. Introduction

Pediatric asthma is the most prevalent chronic respiratory condition in children worldwide and a leading cause of morbidity, school absenteeism, hospitalizations, and healthcare use [1,2,3]. Estimates from the Global Burden of Diseases, Injuries, and Risk Factors study (GBD) 2021 found that over 4% of world’s children and adolescents younger than 20 years suffered from asthma, with higher prevalence being found among males, individuals from 5 to 9 years old and high income groups [2]. The same study highlights an overall decline in asthma prevalence from 1990 to 2021, consistent across most countries and all pediatric age groups.

Asthma is considered an umbrella term that encompasses a group of heterogenous disorders characterized by chronic airway inflammation [4,5]. Such inflammation may cause hyperresponsiveness of the airways, a mucus accumulation, and bronchospasms, resulting in variable limitations to the airflow [5]. This process leads to respiratory symptoms and signals such as dyspnea, shortness of breath, wheezing, chest tightness, and coughing [5,6]. Given its heterogeneity, recent research has been emphasizing the importance of accurately identifying specific phenotypes and endotypes in pediatric patients to better adapt disease management and predict prognosis [7]. Some of the already-identified phenotypes include eosinophilic, allergic and non-allergic, cough variant and cough predominant, asthma with persistent airflow limitation, and asthma associated with obesity [6].

The chronic and heterogeneous nature of pediatric asthma can exert a multidimensional impact on the patients’ lives. From a physical health perspective, asthma’s respiratory manifestations may impose significant limitations on exercise tolerance and participation in physical activities (e.g., playing sports, walking, running) due to symptom triggers, fatigue, or fear of exacerbation [8]. The characteristic chronic airway inflammation of asthma leads to airway hyperresponsiveness, mucus accumulation, and bronchospasm, resulting in variable airflow limitations manifested as dyspnea, wheezing, chest tightness, and coughing [9]. These symptoms can significantly restrict children’s ability to engage in age-appropriate physical activities, contributing to reduced cardiovascular fitness and increased sedentary behavior [9]. In addition, severe cases of asthma are frequently associated with other conditions, such as obstructive sleep apnea and obesity, and may lead to frequent exacerbations and hospitalizations [9,10,11].

Beyond the somatic manifestation, there is increasing evidence of the psychological burden of pediatric asthma. For instance, recent studies have found out that children and adolescents with asthma often present symptoms of anxiety and depression and a personality characterized by shyness and impulsivity [10,12]. This is particularly relevant considering that emotions play an important role in the development of an asthmatic crisis, establishing a bidirectional relationship between psychological well-being and asthma control [12]. The constant concern over potential asthma attacks and the need to manage a chronic condition may contribute to higher stress levels, creating a cycle in which poor asthma control exacerbates anxiety and depression, which in turn may trigger further exacerbations [12].

School and social dimensions can also be significantly impaired by asthma. Children and adolescents with suboptimal asthma control experience school absenteeism more frequently, which impacts their learning process and performance, their participation in social and extracurricular activities, and, therefore, their social development, leading to isolation and difficulty in maintaining peer relationships [8,12,13]. One study also indicates that bullying situations may occur more frequently [12].

The social impact can also extend to family and caregivers. Managing a chronic illness at pediatric age requires vigilance, and asthma control plays an important role, as studies suggest that the caregivers of patients with uncontrolled asthma had significantly lower quality of life than those of patients with controlled asthma [14,15,16,17]. Parents and caregivers face substantial psychological and socioeconomic burdens throughout the long-term caregiving process. Research demonstrates that over one-third of parents experience moderate to high levels of caregiver burden, with 81.52% reporting high parenting stress and psychological distress, 67.57% experiencing varying degrees of depression, and 29% diagnosed with post-traumatic stress disorder [18]. These psychosocial burdens extend to decreased mental health, quality of life, sleep quality, and increased family stress [19]. Another study reviewed the psychological and socioeconomic burdens faced by family caregivers and found decreased indicators of mental health and sleep, family stress, and work and financial challenges [18].

Given the limiting and disruptive impact of asthma on the lives of children and adolescents, several studies found a strong correlation between asthma control and health-related quality of life (HRQoL), where better symptom management translated into improved physical activity, emotional well-being, and social engagement [9,20]. In the context of pediatric asthma, HRQoL refers to the overall impact of the disease on the patient’s physical, emotional, social, and day-to-day functioning [20]. As advances in biomedical science and technology have been improving the survival and disease management of pediatric chronic conditions, HRQoL assessment has become an increasingly relevant outcome measure, shifting focus from purely clinical endpoints to patient-centered outcomes that capture the lived experience of disease [21]. HRQoL assessment in pediatric populations is particularly important given the importance of childhood in the multidimensional development process. Research consistently demonstrates that pediatric patients with asthma report significantly impaired HRQoL compared to healthy children across physical, emotional, school, and social functioning domains, with medium to large effect sizes [21]. Specific tools were developed to measure this construct, such as the Pediatric Asthma Quality of Life Questionnaire (PAQLQ), allowing for a standardized assessment of the patients’ asthma-related quality of life [22].

Promoting adequate treatment for asthma control plays an essential role in promoting HRQoL. Effective disease management hinges on achieving and maintaining a state of minimal symptoms, infrequent exacerbations, no activity limitations, and a near-normal lung function [6]. In that regard, following comprehensive stepped guidelines (e.g., Global Initiative for Asthma–GINA 2024 guidelines) in which treatment intensity is adjusted according to symptom control, exacerbation history, lung function, and HRQoL is of utmost importance [23]. Current guidelines stress a stepwise approach based on pharmacologic and non-pharmacologic (e.g., trigger avoidance, education) interventions. Health education is one of the most significative non-pharmacologic interventions for pediatric patients and their respective families/caregivers, and it generally focus on asthma literacy and practical strategies to manage the disease, promoting adherence to treatment, better asthma control, and increased autonomy in disease management [24,25,26,27,28].

The core pharmacological treatments for pediatric asthma include inhaled corticosteroids (ICS) and short-acting beta-agonists (SABA) for mild cases, and add-on therapies for moderate to severe or uncontrolled cases [6,29]. Among the add-on therapies, options include long-acting beta-agonists (LABA) in combination with ICS, leukotriene receptor antagonists, and, for severe cases, biologic agents targeting specific inflammatory pathways [30,31].

Biologic therapies represent a significant solution for children and adolescents with moderate to severe asthma that is unresponsive to standard treatments. These therapies target specific inflammatory pathways that are determinant to asthma pathophysiology—particularly type 2 inflammation mediated by cytokines such as IL-4, IL-5, IL-13, and IgE [32]. The main biologics currently approved for pediatric use include Omalizumab (anti-IgE), Mepolizumab and Benralizumab (anti-IL-5/IL-5R), Dupilumab (anti-IL-4Rα), and Tezepelumab (anti-TSLP) [31]. Recent clinical trials and real-world studies have demonstrated significant results from biologic therapies in reducing severe asthma exacerbations, decreasing oral corticosteroid use, and improving asthma control and lung function in pediatric patients, especially in those with T2-high endotypes—children who have eosinophilic inflammation or who are allergic [31,32,33,34,35,36].

We are aware of the high costs of biological therapies, but the impact of these therapies has demonstrated enormous effects on the quality of life of children, families, and caregivers, such as a reduction in visits to the emergency room and hospital, a decrease in crises and their severity, better preservation of lung function, and less absenteeism. At the same time, reducing the unpredictability of the disease decreases the psychological and physical burden on caregivers, associated with the constant fear of crises, sleep interruptions, and the impact on daily routine, favoring less anxiety, reduced work absenteeism, and the restoration of family balance. Biological therapy is not only a pharmacological evolution targeting T2 pathways, but also a comprehensive intervention that promotes greater well-being in the family and the child.

The above reasons reinforce the relevance, pertinence, and usefulness of this study. Despite the increasing clinical use of biologic therapies in pediatric patients with moderate to severe asthma, there are few studies that highlight the relationship between these therapies and the quality of life of patients, family members, and caregivers. Therefore, the aim of this systematic review is to study science-based evidence on biologic therapies and their implications for HRQoL in children and adolescents with asthma.

## 2. Materials and Methods

This systematic review was conducted according to the updated guidelines of the Preferred Reporting Items for Systematic Reviews and Meta-analysis (PRISMA) 2020 statement [37]. The protocol for this systematic review was registered on PROSPERO (ID CRD420251112099).

### 2.1. Literature Search Strategy

A systematic literature search was conducted on the PubMed, Scopus, and Web of Science databases using the following keyword string: (asthma) AND (pediatric OR children OR adolescents) AND (biologic therapies OR omalizumab OR mepolizumab OR benralizumab OR dupilumab OR tezepelumab) AND (quality of life OR HRQoL OR PAQLQ). To narrow the search without compromising the results, a field tag was added to every keyword group according to the available options in each database. The keyword “asthma” and the keyword group related to the biologic therapies were searched in the title and abstract fields, while the second and last keyword groups (relating to the population and outcome in study, respectively) were searched in all fields. The following specific search strings for each database were used:

- PubMed: (((asthma[Title/Abstract]) AND ((pediatric OR children OR adolescents)) AND (biologic therapies[Title/Abstract] OR omalizumab[Title/Abstract] OR mepolizumab[Title/Abstract] OR benralizumab[Title/Abstract] OR dupilumab[Title/Abstract] OR tezepelumab[Title/Abstract])) AND ((quality of life OR HRQoL OR PAQLQ));
- Scopus: TITLE-ABS-KEY(asthma) AND ALL(pediatric OR children OR adolescents) AND TITLE-ABS-KEY(biologic therapies OR omalizumab OR mepolizumab OR benralizumab OR dupilumab OR tezepelumab) AND ALL(quality of life OR HRQoL OR PAQLQ);
- Web of science: (((TS=(asthma)) AND ALL=(pediatric OR children OR adolescents)) AND TS=(biologic therapies OR omalizumab OR mepolizumab OR benralizumab OR dupilumab OR tezepelumab)) AND ALL=(quality of life OR HRQoL OR PAQLQ).

Specific filters were used to restrict language and publication date. As such, the search was focused on articles written in English, Portuguese, or Spanish published between 2015 and 2025. This ten-year period was selected after a preliminary search using the same databases and search strings of the search strategy, covering the most recent and significant scientific production on this line of research. This preliminary search allowed the authors to verify a significant and unprecedented development and dissemination in the last 10 years, reinforcing our interest in this time frame. This criterion is also backed by existing literature regarding the inclusion of search date limits in literature reviews, including systematic reviews [38,39]. The increase in literature publication during this period leads to the necessity of compiling the most recent evidence, allowing for the evolution and dissemination of knowledge in this area.

### 2.2. Study Selection Criteria

Database search results were exported to Zotero (version 7.0.22), a reference management software, in which duplicates were detected and manually removed. The remaining records were included in the screening process, in which eligibility criteria were implemented. The definition of this criteria was based on the PICOS methodology, ensuring the selected articles were relevant to the research question [40].

#### 2.2.1. (P) Population

Pediatric patients aged below 18 years old, diagnosed with asthma according to established guidelines (e.g., GINA guidelines) [41]. All phenotypes of asthma were considered eligible.

#### 2.2.2. (I) Intervention

The main biologic therapies currently used in asthma patients were considered, namely Omalizumab, Mepolizumab, Benralizumab, Dupilumab, and Tezepelumab. No restrictions were considered regarding their administration (i.e., alone or combined, dosage, duration).

#### 2.2.3. (C) Comparison

No restrictions were considered on this topic. Comparisons with standard treatments, placebos, other biological therapies, or studies with no comparison were considered.

#### 2.2.4. (O) Outcomes

Quality of life as an outcome related to asthma and the administration of biologic therapies. Only studies reporting quantitative data from standardized QoL questionnaires were considered.

#### 2.2.5. (S) Types of Studies

Systematic reviews, meta-analyses, editorials, single case reports, literature reviews, and letters were excluded.

### 2.3. Data Extraction Process

The articles that resulted from the screening process were analyzed individually and relevant information was extracted. This process was conducted by three reviewers independently after a standardized spreadsheet was developed and the reviewers discussed and compared the results to ensure that a consensual result was obtained [42]. Discrepancies in data extraction were discussed until a consensus could be reached between the three reviewers.

Data extraction included the following information:

- Name of the first author and year of publication;
- Countries in which the study was conducted;
- Type of study;
- Number of participants, their age range, and gender;
- Condition (i.e., asthma phenotype);
- Biologic therapy administered to the participants;
- Comparison between groups (e.g., biologic vs. placebo);
- Treatment and follow-up duration;
- QoL instrument and version used;
- Treatment efficacy on asthma control;
- QoL outcomes.

These topics were selected to allow for a comprehensive analysis of the included studies. Due to methodological differences, some topics do not apply to all the articles.

### 2.4. Study Quality Assessment

Given that the selection criteria allowed the inclusion of different types of studies, a structured risk of bias (RoB) assessment was conducted for all studies using the Mixed Methods Appraisal Tool (MMAT)—version 2018 [43]. This tool was selected for its ability to evaluate diverse methodologies with a unified framework, allowing for harmonized RoB assessment. MMAT appraises the quality of empirical studies and comprises two general screening questions, which indicate if the study can be appraised using the MMAT, and five methodological criteria for each study design category: qualitative; quantitative randomized controlled trials; quantitative non-randomized; quantitative descriptive; and mixed methods. Compliance with all five criteria suggests good methodological quality.

The assessment was carried out independently by three researchers and the results were compared so that a consensus could be reached.

## 3. Results

### 3.1. Literature Identification and Screening

**Figure 1:**
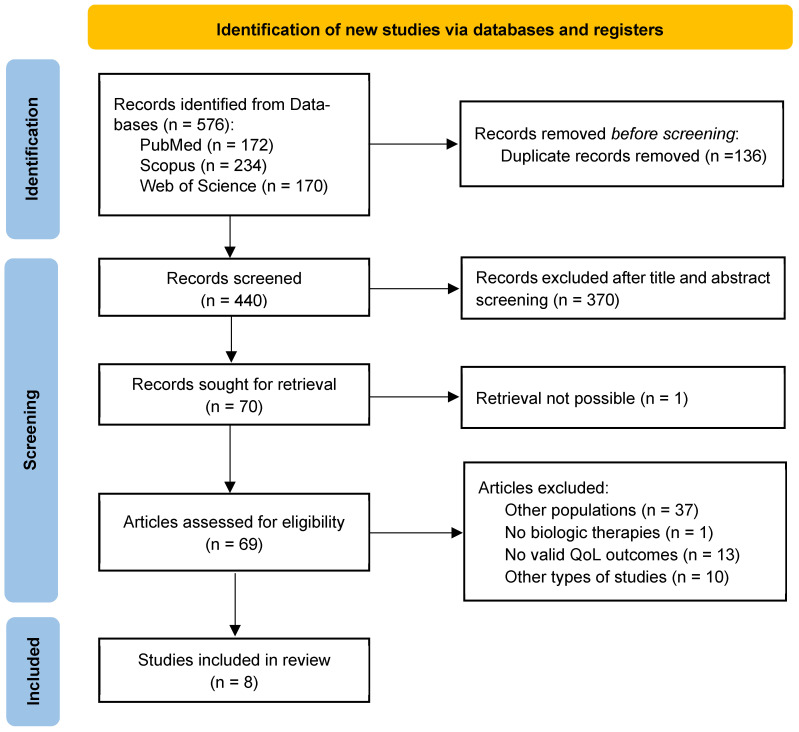
PRISMA flow diagram.

The search strategy resulted in a total of 576 records before duplicate removal, as illustrated in Figure 1. After 136 duplicates were identified and removed, the titles and abstracts of 440 records were screened following the PICOS methodology, and 370 did not meet the selection criteria. In this phase, titles/abstracts that did not provide enough information to reach a conclusive screening decision were conditionally accepted for retrieval. Only one record was not retrievable since the full text was not available.

Assessment for eligibility was completed for 69 articles, of which 61 were excluded mainly because of their population, intervention, outcome, or type of study. Regarding the population, 37 studies included only adults (n = 24) or both pediatric and adult individuals (n = 12) but did not present specific results for pediatric individuals [44,45,46,47,48,49,50,51,52,53,54,55]. One article did not fit the intervention criteria previously described [56]. Regarding the outcome, 13 articles were excluded because QoL was not an outcome, or it was presented as an outcome even though it was not related to asthma, because no data was available, or because the results were based on the literature regarding the relationship between symptom frequency and QoL but without a specific instrument having been administered [57,58,59,60,61,62,63,64,65,66,67,68,69]. As for the type of study, ten study protocols, editorials, single case reports, literature reviews, and letters were excluded [70,71,72,73,74,75,76,77,78,79]. After the screening process, eight articles were selected.

### 3.2. Study Characteristics

Eight studies complied with the established selection criteria and were included in this review. The articles were published between 2015 and 2025, and study designs were diverse, including one randomized controlled trial (RCT), one mixed-methods, six quantitative non-randomized, and one quantitative descriptive.

These studies involved pediatric patients with moderate to severe asthma who were treated with biologic therapies, in particular Omalizumab, Dupilumab, and Mepolizumab. Despite Tezepelumab also being considered during the search and screening processes, no articles about this therapy complying with the criteria were found. The main characteristics of the studies are presented in Table 1.

The methodological quality assessment data is presented in Table 2. Overall, the studies met the established category-specific methodological quality criteria. In the RCT, participants were allocated into intervention or control groups in an appropriate randomized manner, and all the participants adhered to the assigned interventions and contributed to all the measures, enabling the authors to present complete outcome data, and the researchers assessed the outcomes following a double-blind approach, avoiding methodological biases. In the quantitative non-randomized studies, most studies presented participants representative of the target population, the selection of measurements regarding the outcome and intervention were considered appropriate, and the intervention/exposure was administered as intended. The main identified issue relates to the consideration of confounding variables in the study design analysis, which may have led to bias in the interpretation of the results. Regarding the quantitative descriptive study, the sampling strategy used by the researchers was considered relevant to address the proposed research question, and the sample was representative of the target population. In addition, the selected measurements were deemed appropriate and the participants’ responses and the statistical analysis seemed appropriate to answer the research question.

### 3.3. Efficacy of Biologic Therapies on Asthma Control and HRQoL

All the selected studies demonstrated the significant efficacy of biologic therapies—omalizumab, mepolizumab, and dupilumab—in improving asthma control and reducing symptomatology and corticosteroid use in pediatric patients with moderate to severe or uncontrolled asthma.

Regarding biologic therapies, six articles studied the impact of omalizumab, three studied the impact of dupilumab and just one included mepolizumab. Despite significant differences in study design and methodology across the articles, particularly in terms of assessment strategy (e.g., cross-sectional vs. longitudinal, prospective vs. retrospective) and duration of treatment and follow-up, symptom control assessments using tools like the Asthma Control Test [88] showed clinically significant improvements after biologic therapy initiation. Patients report fewer daytime and nighttime symptoms, leading to better overall asthma control scores and quality of life, as measured by PAQLQ or other QoL assessment tools (Table 3). Additionally, corticosteroid use for exacerbation control was substantially diminished, lowering the risk of steroid-related adverse effects [87].

Safety profiles across pediatric studies show that biologics are generally well tolerated, with mild adverse events such as injection site reactions and transient upper respiratory symptoms being the most common. No significant new safety concerns in children emerged within treatment durations of up to one year.

The positive impact of biologic therapies seems to extend to HRQoL. In studies where comparisons were made between biologic therapy use and non-use, significant asthma-related QoL improvements were found. These improvements were mostly assessed using PAQLQ or a specific version of the instrument, as shown in Table 4. Additionally, one study compared asthma-related QoL between omalizumab, mepolizumab, and dupilumab, and found no significant differences between groups, suggesting equal impact between the three therapies. In addition to pediatric patients, two studies also assessed the impact of dupilumab and omalizumab on the QoL of caregivers [80,86]. Both studies presented significant improvements in overall QoL, activity limitations, and emotional function.

#### 3.3.1. Dupilumab and HRQoL

Results from the VOYAGE study [80] and Herzig et al. (2025) [81] presented significant HRQoL improvements when administering Dupilumab for pediatric asthma. The VOYAGE study [80] found that the treatment led to a global increase in PAQLQ(S)-IA scores from week 24 onwards, particularly in emotional function, activity limitation, and symptoms, which was supported by a sustained reduction in asthma symptoms and increased asthma control. Herzig et al. (2025) [81] reported a median of 6.3 in the PAQLQ(S) questionnaire in patients who were taking dupilumab, suggesting a low impact of the patients’ asthma on their quality of life when taking this biologic therapy.

In addition to patients’ quality of life, the VOYAGE study also analyzed the caregivers’ QoL. Reported results indicated a significant improvement in Pediatric Asthma Caregiver Quality of Life Questionnaire (PACQLQ), particularly in the dimensions of emotional function and activity limitation.

#### 3.3.2. Omalizumab and HRQoL

Results from Lu et al. (2025) [83], Odajima et al. (2025) [84], Su et al. (2023) [85], Sztafinska et al. (2017) [86], and Sztafinska et al. (2021) [87] are in line with the HRQoL improvements found for the Dupilumab treatment. The study of Odajima et al. (2025) [84] found a significant increase in QoL short form version 2008 (Gifu) scores in both children aged 6 to 15 and their parents or caregivers, particularly in physical and emotional domains. Likewise, Sztafinska et al. (2017) [86] found significant score improvements in all domains of the PAQLQ and PACQLQ for patients and parents/caregivers, respectively. Such results were considered stable across the time of the study (measured at 16, 52, and 104 weeks after treatment) and a positive correlation was found between patients’ HRQoL improvement and caregiver QoL. In the study of Su et al. (2023) [85], the sample (aged from 6 to 18 years) was divided into two groups: an overall group, including the entire sample; and a group of pediatric patients from 6 to 12 years of age. The results suggested similar improvements in PAQLQ scores 24 weeks after the treatment for both groups, reporting an average effect of omalizumab on patient’s HRQoL. Sztafinska et al. (2021) [87] also reported significant improvements in HRQoL after one year of treatment, as reported by the mini-AQLQ scores. Following the same pattern, Lu et al. (2025) [83] assessed the impact of Omalizumab in children from 6 to 14 years of age and found that the PAQLQ scores improved after 16 weeks of treatment.

#### 3.3.3. Mepolizumab and HRQoL

Only one of the selected studies addressed the impact of mepolizumab on the patients’ HRQoL. The SPACE study [82] compared the impact of omalizumab, mepolizumab, and dupilumab in pediatric asthma patients, having reported a median score of 6.3 in PAQLQ, which suggests a low impact of the disease on HRQoL.

### 3.4. Studies’ Main Limitations

The selected studies present some limitations related to their methodology, population, and/or study design.

In terms of the study design and sampling, some of the studies [82,85,86,87] point to the absence of a control group, using an uncontrolled, open, single-arm design, not allowing for a direct comparison with a group of patients not treated with biologics. Particularly in Sztafinska et al. (2017) [86], since all the patients who fulfilled criteria for anti-IgE treatment were treated with omalizumab, a control group could not be identified and used for comparison. Another limitation presented by Herzig et al. (2025) [81] refers to the size of the sample, which encourages a cautious interpretation of the results and hinders a generalization of the results. A cross-sectional study design, as used in some of the studies [81,82], also hinders the accuracy when assessing the impact of biologic therapies on HRQoL before, during, and after treatment. Regarding the population, the VOYAGE study [80] included a relatively homogenous population, including mostly white children, hindering a generalization of the results to ethnically diverse populations.

Another limitation of the studies relates to their assessment of HRQoL. Some hindering factors related to an assessment based on patient-reported outcomes were pointed out by the studies, including the age of the children and their limited capacity to assess subjective criteria, which may have affected the reliability of the reports, the role of the parents during the assessment, which may have conditioned the children’s reports, and the lack of consideration for the contexts in which the children experience their disease (e.g., at school, during play time, during after-school activities). In the VOYAGE study [80], the HRQoL questionnaire was administered by an interviewer, which may have impacted the answers of the children.

If we take into consideration the specific factors related to the treatment, some limitations were also considered by the studies. For example, Liu et al. (2025) [82] point to the need for further clarification about the definition and possibility of disease remission, long-term strategies for disease management in patients that achieved good control with biologic treatments, and if/when the treatment with biologics can be discontinued and how to adjust other treatments. In the PASS study [85], another limitation refers to the adherence to treatment, since part of the sample of this study pointed out the financial costs as a significant limitation to starting treatment with biologics.

## 4. Discussion

This systematic review aimed to study science-based evidence on biologic therapies and its implications for HRQoL in children and adolescents with asthma. The findings consistently support the fundamental role of biologic agents—particularly omalizumab, mepolizumab, and dupilumab—in improving asthma control and promoting patient health-related QoL in patients with moderate to severe or uncontrolled asthma. All of the studies reported statistically significant improvements in HRQoL scores, with patients consistently achieving median scores on the HRQoL scales, indicating minimal disease impact on quality of life after biologic therapy initiation [80,81,82,83,84,85,86,87]. These changes were observed in studies with different methodological designs and across different geographic regions and patient populations, suggesting a beneficial impact of omalizumab, mepolizumab, and dupilumab in pediatric asthma HRQoL promotion and disease management.

The results highlighted the significant impact of biologic therapies on pediatric HRQoL in children/adolescents and their families/caregivers. A noticeable increase in scores of asthma-related QoL was found across all studies, which, aligned with the results of asthma control, reinforces the previously reported relationships between asthma control and psychological well-being and HRQoL [9,12,20]. Two studies also found significant improvements in caregivers’ QoL, which, associated with the efficacy of biologic therapies in improving asthma control and HRQoL in pediatric patients, is in line with previous studies suggesting a relationship between controlled/uncontrolled asthma and caregivers’ QoL [14,15,18]. The included studies also reported significant reductions in symptom burden, oral or inhaled corticosteroid use, asthma exacerbation, and hospitalization, with variable periods of therapy administration, being in line with previous studies that assessed the efficacy of biologic agents on pediatric asthma, particularly on type 2 (T2)-high-inflammatory phenotypes [31,32,33,91].

Prior reviews of biologic therapies have largely concentrated on clinical metrics such as FEV_1_ improvements, exacerbation reduction, and systemic steroid-sparing effects [31,33]. However, few have centered their analyses on QoL as a primary outcome. This review addresses that gap, reaffirming the centrality of HRQoL in pediatric asthma care and offering a broader and more integrative understanding of biologic efficacy.

Our findings underscore the broader therapeutic benefits of biologic therapies, extending beyond disease control to impact the lived experiences of both pediatric patients and their families. Pediatric asthma exerts a multifaceted impact that spans physical health, emotional well-being, social functioning, academic performance, and family dynamics [8,9,10,11,12,13,14,15,18]. Understanding these dimensions is critical to comprehensive care that addresses not only the symptoms but also the full burden of the disease on children and their caregivers.

### 4.1. Mechanisms Linking Biologic Therapies to HRQoL Improvements

The significant improvements in HRQoL observed across all included studies may be understood through multiple interconnected mechanisms that extend beyond simple symptom reduction. At the physiological level, biologic therapies contribute to the reduction in type 2 inflammation by targeting specific cytokines (IL-4, IL-5, IL-13) and IgE, leading to decreased airway hyperresponsiveness, reduced mucus production, and improved lung function [34,92,93,94]. This translates into fewer respiratory symptoms, such as less dyspnea, wheezing, and coughing, enabling children to engage more fully in physical activities such as sports, play, and exercise without fear of triggering exacerbations.

At a psychological level, the reduction in symptom burden and exacerbation frequency may foster an increased sense of control and self-efficacy in managing their disease [8,9,10,11,12,13,14,15,17,35]. Children and adolescents who experience consistent asthma control through biologic therapy may develop greater confidence in their ability to participate in activities, reduced anticipatory anxiety about asthma attacks, and experience improvements in overall emotional well-being [17]. The bidirectional relationship between psychological well-being and asthma control, where emotional distress can trigger exacerbations and poor asthma control increases anxiety and depression, suggests that biologic therapies may interrupt this negative cycle, creating a positive feedback loop of improved control and enhanced mental health.

In the social dimension, improved asthma control enables greater school attendance and participation in social and extracurricular activities [8,9,17]. Reduction in absenteeism supports academic performance and allows children to maintain peer relationships and social development, areas often disrupted in children with uncontrolled asthma.

### 4.2. Parents and Caregivers’ QoL

Two studies in this review demonstrated significant improvements in parents and caregivers’ QoL, suggesting that biologic therapies may have a family-level impact too [80,86]. When parents and caregivers experience reduced stress, improved sleep, and fewer disruptions to work and family routines due to their child’s improved asthma control, it may further enhance the child’s emotional and social environment [16,17], creating additional indirect benefits for the child’s HRQoL.

### 4.3. Comparative Effectiveness Across Biologic Agents

An important finding of Liu et al. (2025) [82] refers to the comparison between omalizumab (anti-IgE), mepolizumab (anti-IL-5), and dupilumab (anti-IL-4Rα). Despite being based on different specific mechanisms of action, no significant differences in HRQoL outcomes were found between them, with all achieving median PAQLQ scores of 6.3. This suggests similar potential of these biologics in promoting patient-centered outcomes, even though they target distinct inflammatory pathways.

Such findings have important clinical implications. We may hypothesize that when biologics are appropriately matched to patient phenotypes and endotypes (e.g., allergic asthma for omalizumab, eosinophilic asthma for mepolizumab, type 2 inflammation for dupilumab), they may produce comparable improvements in HRQoL despite differences in their mechanisms of action, supporting a “treat-to-target HRQoL” approach, in which clinicians select biologics based on patient-specific biomarkers and phenotypes, with the expectation that adequate disease control will translate into similar HRQoL gains across different agents [95]. However, such findings should be interpreted cautiously. The study of Liu et al. (2025) [82] employed a cross-sectional design without baseline HRQoL assessment, and patients were not randomized into biologic groups, but rather, treatment selection was based on clinical characteristics and biomarker profiles. Therefore, differences in baseline disease severity, phenotype distribution, or treatment duration across groups may have biased possible differences in biologic efficacy.

### 4.4. Strengths and Limitations of This Review

#### 4.4.1. Strengths

This systematic review presents several methodological strengths that enhance the reliability and comprehensiveness of our findings. First, we employed a rigorous and comprehensive search strategy across three major databases (PubMed, Scopus, and Web of Science), ensuring broad coverage of the available literature on biologic therapies and HRQoL in pediatric asthma. Second, our review adhered strictly to PRISMA 2020 guidelines, with a registered protocol on PROSPERO (ID CRD420251112099), ensuring transparency and methodological rigor throughout the review process. Third, we used three independent reviewers’ assessments for study selection, data extraction, and quality appraisal, minimizing selection bias and ensuring consensus on all decisions. Fourth, we applied the Mixed Methods Appraisal Tool (MMAT), a validated and flexible instrument that allowed us to assess the methodological quality of diverse study designs within a unified framework.

Furthermore, this review addresses a critical gap in the existing literature by focusing specifically on HRQoL as a primary outcome, rather than solely on clinical endpoints such as lung function or exacerbation rates. By synthesizing evidence on patient-centered outcomes, this review provides valuable insights into the multidimensional impact of biologic therapies on the lived experiences of children, adolescents, and their families. Additionally, our inclusion of diverse study designs (RCTs, prospective cohorts, retrospective observational studies, and cross-sectional studies) enhances the real-world applicability of our findings, reflecting the heterogeneity of clinical practice settings.

#### 4.4.2. Limitations

Despite these strengths, several limitations must be acknowledged. First, although we applied a comprehensive search strategy, the use of publication date (2015–2025) and language filters (English, Portuguese, Spanish) may have excluded relevant studies published earlier or in other languages, potentially limiting the breadth of our review. Second, only eight studies met our inclusion criteria, reflecting the limited volume of published research specifically examining HRQoL outcomes in pediatric biologic therapy users. This small evidence base, combined with considerable methodological heterogeneity across studies, precluded the possibility of conducting a meta-analysis to quantitatively synthesize effect sizes. Third, the included studies exhibited geographic clustering, with a predominance of research conducted in Europe and Asia. This limits the generalizability of findings to other populations, particularly children and adolescents from low- and middle-income countries, ethnic minorities, and regions with different healthcare systems and access to biologic therapies. Fourth, publication bias remains a concern, as we only included published peer-reviewed studies and did not search gray literature or trial registries for unpublished data. It is possible that studies with null or negative findings on HRQoL outcomes were less likely to be published, which could inflate the observed treatment effects in this review.

### 4.5. Future Research Directions

Despite the relevance of the findings, it is important to acknowledge existing research gaps. Most studies currently published present moderate to small size samples, limited representation of different asthma phenotypes and endotypes, and short to medium durations of treatment and/or follow-up. Further studies are needed to verify the long-term multidimensional impacts of administering biologic therapies to children and adolescents with moderate to severe asthma, specifying for each biologic agent and for specific phenotypes, endotypes, and patients with associated comorbidities. As suggested by recent scientific articles, such impacts may be crucial to better address disease management and patient empowerment [28,36]. The underrepresentation of certain biologics (such as tezepelumab and benralizumab) as well as the potential impact of socioeconomic barriers to biologic access and broader healthcare disparities, should be addressed in the future. Longitudinal studies are also needed to assess the long-term HRQoL outcomes in patients and their respective families and caregivers. Standardization of asthma-related QoL assessment tools and consistent reporting will further strengthen the evidence base and facilitate meta-analyses. Also, there are few articles that highlight the use of specific contextual guidelines in biological therapies, which we consider to be very important. Based on international guidelines (Gaillard & Moelle, 2021) [96], we suggest that specific guidelines for the Portuguese context could be developed.

## 5. Conclusions

Biologic therapies can play an important role in promoting the quality of life of children/adolescents with moderate to severe asthma and their families/caregivers. These therapies provide a significant reduction in exacerbations, hospitalizations and the need for systemic corticosteroids, allowing for better symptom control and greater stability of respiratory function. Despite their potential in promoting the multidimensional well-being of both patients and caregivers, adoption is still limited, partially due to the costs associated with this form of treatment. In addition to showing the advantages of biological therapies, this study also warns that greater importance should be given to this topic in public health policies. Thus, this study emphasizes the need for greater investment in children and adolescents, with greater optimization of synergies at the level of integrated healthcare in various areas of healthcare, such as medicine, nursing, psychology, and pharmacology, among others. Public health policies should focus on strategies that facilitate greater involvement not only of children and adolescents, but also of families, schools, and the community. The promotion of the quality of life of the pediatric population, in addition to a greater focus on health literacy (e.g., specific knowledge about asthma, nutrition, promotion of healthy lifestyles), should also reinforce education for health and well-being and for self-care, not only for children and adolescents but also for families and caregivers.

## Figures and Tables

**Table 1:** Characteristics of the selected studies.

Authors (Year)	Countries Involved	Study Design	Population			Intervention		
			Number of Participants	Age (Years)	Condition	Biologic	**Duration**	**Follow-Up**
Fiocchi et al. (2023) [80]	Multinational	RCT (phase 3)	350	6–11	Moderate-to-severe type 2 asthma	Dupilumab	52 weeks	After 24 and 52 weeks
Herzig et al. (2025) [81]	Germany	Non-interventional, cross-sectional, explorative, monocentric	36	6–16	Severe asthma (with or without atopic dermatitis)	Dupilumab	Duration of treatment not specified	No follow-up
Liu et al. (2025) [82]	Europe	Cross-sectional observational	250	6–17	Severe asthma	Omalizumab Mepolizumab Dupilumab	Duration of treatment not specified	No follow-up
Lu et al. (2025) [83]	China	Prospective cohort, single-arm trial	43	6–14	Moderate to severe asthma	Omalizumab	16 weeks	Every 4 weeks
Odajima et al. (2015) [84]	Japan	Prospective clinical, open-label	38	6–15	Severe allergic asthma	Omalizumab	24 weeks	16 weeks
Su et al. (2023) [85]	China	Retrospective observational cohort	316 *	6–18	Moderate to severe allergic asthma	Omalizumab	Duration of treatment not specified	24 weeks
Sztafińska et al. (2017) [86]	Poland	Prospective cohort	19	6–15	Severe allergic asthma	Omalizumab	Duration of treatment not specified	104 weeks
Sztafińska et al. (2021) [87]	Poland	Prospective case series	17	8–16	Uncontrolled allergic asthma	Omalizumab	1–3 year	Up to 4, 5 years

* Note: the presented number refers to the subgroup of participants of less than 18 years old.

**Table 2:** Study quality assessment using the MMAT—version 2018.

MMAT Category	Study	Methodological Quality Criteria						
		S1 *	S2 *	Q1 *	Q2 *	Q3 *	Q4 *	Q5 *
Quantitative RCT	Fiocchi et al. (2023) [80]	Yes	Yes	Yes	Yes	Yes	Yes	Yes
Quantitative non-randomized	Herzig et al. (2025) [81]	Yes	Yes	Yes	Yes	Yes	Cannot tell	Yes
	Liu et al. (2025) [82]	Yes	Yes	Yes	Yes	Yes	Yes	Yes
	Lu et al. (2025) [83]	Yes	Yes	Yes	Yes	Yes	Cannot tell	Yes
	Odajima et al. (2015) [84]	Yes	Yes	Yes	Yes	Yes	No	Yes
	Su et al. (2023) [85]	Yes	Yes	Yes	Yes	Yes	Cannot tell	Yes
	Sztafińska et al. (2017) [86]	Yes	Yes	Yes	Yes	Yes	No	Yes
Quantitative descriptive	Sztafińska et al. (2021) [87]	Yes	Yes	Yes	Yes	Yes	Yes	Yes

* Note: S1 and S2 refer to the general screening questions (common for all types of studies), while Q1 to Q5 refer to the specific methodological criteria for each category of study design; S1—research question clarity; for the specific questions, please refer to correspondent study design in the MMAT version 2018 [32].

**Table 3:** Main results of the selected studies.

Study	Biologic	Asthma Control	HRQoL
Fiocchi et al. (2023) [80]	Dupilumab	Significant longitudinal improvements in patients with type 2 phenotype.	Significant improvement in pediatric asthma-related QoL in dupilumab treated patients compared to placebo
Herzig et al. (2025) [81]	Dupilumab	Significant patient-reported improvements in disease management.	Dupilumab users presented better QoL scores
Liu et al. (2025) [82]	Omalizumab Mepolizumab Dupilumab	Most patients obtained good symptom control, but still experienced asthma.	No significant differences found between biologic therapy groups
Lu et al. (2025) [83]	Omalizumab	Patients’ asthma control scores improved after 1 week of treatment; reduced ICS and LABA use; reduced frequency of acute asthma exacerbations.	Significant improvements in asthma-related QoL scores after treatment
Odajima et al. (2015) [84]	Omalizumab	Significant improvements in total asthma symptoms, daily activity and nocturnal sleep scores; significant reduction in asthma exacerbation and hospitalization; some patients still experienced at least one adverse event during treatment.	Significant asthma-related QoL improvements between baseline and week 24 of treatment
Su et al. (2023) [85]	Omalizumab	Treatment improved lung function and asthma control; only a minority of patients reported adverse events and serious adverse events.	Significant improvements in asthma-related QoL in all domains and overall scores
Sztafińska et al. (2017) [86]	Omalizumab	Treatment was associated with reduction in ICS use	Significant improvements in asthma-related QoL scores
Sztafińska et al. (2021) [87]	Omalizumab	Improvements in asthma control, reduction in ICS and oral corticosteroids use, and decreased exacerbations	Significant increase in asthma-related QoL scores

**Table 4:** Instruments of the selected studies.

Instrument	Studies	Instrument Characteristics			
		Number of Items	Domains/Components	Scoring	Score Range
PAQLQ/ PAQLQ(S) */ PAQLQ(S)-IA * [22]	Fiocchi et al. (2023) [80] Herzig et al. (2025) [81] Liu et al. (2025) [82] Lu et al. (2025) [83] Su et al. (2023) [85] Sztafińska et al. (2017) [86]	23	Symptoms Activity limitation Emotional function	1 to 7 (Higher score = better QoL)	23–161
miniAQLQ [89]	Sztafińska et al. (2021) [87]	15	Symptoms Environmental stimuli Emotional function Activity limitation	1 to 7 (Higher score = better QoL)	15–105
QoL short form version 2008 (Gifu) [90]	Odajima et al. (2015) [84]	10	Physical domain (asthma attack; instability of symptoms; exercise load) Emotional domain (emotional burden; proper acceptance of asthma)	1 to 5 (1 = Severe impairment; 5 = No impairment)	10–50

* Note: (S)–Standardized version; (IA)—Interviewer-Administered.

## Data Availability

All data used in this study are available in the manuscript.

## Abbreviations

The following abbreviations are used in this manuscript:

GINA	Global Initiative for Asthma
HRQoL	Health-Related Quality of Life
ICS	Inhaled corticosteroids
LABA	Long-acting beta-agonists
MMAT	Mixed Methods Appraisal Tool
PAQLQ	Pediatric Asthma Quality of Life Questionnaire
PRISMA	Preferred Reporting Items for Systematic Reviews and Meta-analysis
QoL	Quality of Life
RCT	Randomized Controlled Trial
RoB	Risk of Bias
SABA	Short-acting beta-agonists
